# Biofunctional Characteristics of Lignite Fly Ash Modified by Humates: A New Soil Conditioner

**DOI:** 10.1155/2010/457964

**Published:** 2010-05-31

**Authors:** Konstantinos Chassapis, Maria Roulia, Evangelia Vrettou, Despina Fili, Monica Zervaki

**Affiliations:** ^1^Inorganic Chemistry Laboratory, Department of Chemistry, University of Athens, Panepistimiopolis, 157 71 Athens, Greece; ^2^School of Chemical Engineering, National Technical University of Athens, 9 Heroon Polytechniou, Zografou Campus, 157 73 Athens, Greece

## Abstract

Fly ash superficially modified with humic substances from the Megalopolis lignitic power plant was prepared and evaluated for agricultural uses. UV-vis spectrophotometry and IR spectroscopy revealed that fly ash shows high sorption efficiency towards humic substances. Adsorption proceeds stepwise via strong Coulombic and hydrophophic forces of attraction between guest and host materials. Langmuir, Freundlich, BET, Harkins-Jura, and Dubinin-Radushkevich isotherm models were employed to evaluate the ongoing adsorption and shed light to the physicochemical properties of the sorbent-adsorbate system. Humic substances desorption and microbial cultivation experiments were also carried out to examine the regeneration of the humates under washing and explore the possibility of this material acclimatizing in real soil conditions, both useful for biofunctional agricultural applications.

## 1. Introduction

Fly ash is an amorphous mixture of ferroaluminosilicate minerals generated from the combustion of ground or powdered coal at 400–1500°C and belongs to the coal combustion by-products in power plants produced from bituminous, subbituminus, and lignite combustion. Fly ash is the mineral residue consisting of small particles that are carried up and out of the boiler in the flow of exhaust gases and are collected from the stack gases using electrostatic precipitators, flue gas desulphurization systems, and bag houses [1]. Approximately 70% of the by-product is fly ash collected in electrostatic precipitators, which is the most difficult to handle [2]. This fact pinpoints the necessity for environment-friendly uses of fly ash. Fly ash is mostly used as a substitute for Portland cement in manufacturing roofing tiles and as structural fill, sheetrock, agricultural fertilizer, and soil amendment [3, 4]. Chemically, 90%–99% of fly ash is comprised of Si, Al, Fe, Ca, Mg, Na, and K with Si and Al forming the major matrix. The mineralogical, physical, and chemical properties of fly ash depend on the nature of parent coal [5, 6]. All these applications are based on the presence of basic mineral elements resembling earth's crust, which makes them excellent substituent for natural materials.

The Greek peaty lignite of the Megalopolis Basin, formed during the Quaternary period and comprising significant quantities of humic substances and inorganic content [7, 8], may be an effective raw material for obtaining both humic substances and fly ash. During the last fifty years, Megalopolis lignite has been almost solely utilized for power generation producing solid wastes such as fly ash, bottom ash, boiler slag, and flue gas desulphurization materials, which have been commonly treated as wastes.

Agricultural utilization of fly ash has been originally proposed mostly thanks to its considerable K, Ca, Mg, S, and P contents [5, 9–11]. It was also realized that fly ash addition could also decrease the bulk density of soils, which, in turn, improved soil porosity and workability and enhanced water retention capacity [9]. Additionally, acidic or alkaline fly ash, may be of agronomic benefit buffering the soil pH [5, 12, 13] and improving the soil nutrient status, thus increasing plant growth and nutrient uptake [14, 15]. The electrical conductivity of soil also increases with fly ash application and so does the metal content. Mixtures of swine manure with fly ash proved to increase the availability of Ca and Mg balancing the ratio between monovalent and bivalent cations, which otherwise proves detrimental to the soil [16]. This is of major importance as the presence of Ca can enhance flocculation or aggregation of soil particles, particularly clay, keeping soil friable, thus allowing both water and roots to penetrate hard and compact soil layers.

Compared to traditional soil-conditioning materials as asbestos, fly ash seems more advantageous as it is an environmentally safe material, contains plant nutrients, and can be used in biological cultivations (EU dir 889/08). Additionally, it is a low-cost material and, thanks to its granular composition, is readily applicable.

Humic substances are natural organic matter ubiquitous in water, soil, and sediments produced from the decay of animal and plant tissues and/or microbial activity. They are multifunctional amorphous biopolymers composed of hundreds of organic constituents including carbohydrates and condensed aromatic rings substituted by carboxylic, phenolic, and methoxyl groups [17], which may serve as an ideal ligand for bioinorganic applications. Their role in soil environment and their contribution in sustaining plant growth are of significant importance. Soil structure (water retention, texture, and workability), biological activity, and sequestration (chemical bioavailability, bioaccumulation, and transport of nutrients) are properties and processes of the global ecosystem directly affected by humic substances. The major challenge in research arises from the extended diversity of humic substances due to their large chemical heterogeneity and geographical variability [18].

Until now, fly ash has been used only as humic acid adsorbent for waste treatment [19, 20]. Therefore, the possibility of producing a material combining both the humic substances and fly ash advantages would appear particularly attractive.

In this work, fly ash superficially modified with humic substances (both derived from the Greek peaty lignite of the Megalopolis Basin) was prepared. UV-vis spectrophotometry and IR spectroscopy were employed to characterize the adsorption process. Five adsorption models were selected to envisage the physicochemical properties of the sorbent-adsorbate system based on the fundamental considerations and theoretical hypotheses of the specific isotherms. Humic substances desorption experiments were also carried out to determine the release rate of the humates adsorbed under washing. Additionally, cultivation experiments of microorganisms were processed to evaluate the possibility of acclimatizing this material in real soil conditions, useful for biofunctional agricultural applications.

## 2. Materials and Methods

Both humic substances and fly ash were extracted from the Greek peaty lignite of the Megalopolis Basin, Greece. The selection of lignite was based on the rich content of humic substances in the particular lignite field [7, 8, 17]. Fly ash is a by-product during lignite combustion in Megalopolis power plants. Samples of fly ash were also received from the Ptolemais Basin (Kardia) for comparison purpose.

The extraction of humic substances from Megalopolis lignite using KOH solutions has been described previously as humates originating from low-rank coals are almost completely soluble in aqueous alkali [17]. A Varian Cary 3E UV-vis spectrophotometer was used to estimate the concentrations of humic substances at *λ*_max _ = 550 nm. Prior to calculations a linear calibration curve was established by plotting absorbance against humic substances content.

In order to investigate the adsorption of humic substances on fly ash the following procedure was applied: 50 mL of humic substances solution were mixed with 1 g fly ash. The mixture was left under stirring for 1 hour at ambient temperature (291 K) and constant pH and then filtered to separate the humates. The concentration of humic substances in the solution was determined spectrophotometrically as described above and the retention of the adsorbate onto adsorbent (mg g^−1^) was calculated by the following equation:

(1) $$Q_{e}=\frac{\left(C_{0}-C_{e}\right)}{m}V,$$

where *Q*_*e*_ is the equilibrium concentration of humic substances on the adsorbent (mg g^−1^), *C*_0_ their initial concentration in solution (mg L^−1^), *C*_*e*_ the equilibrium concentration of humic substances in solution (mg L^−1^), *m* the mass of adsorbent (g), and *V* the volume of the humic substances solution (L).

Adsorption experiments were also repeated at 308, 323, 338, and 353 K and at several pH values to study the effect of temperature and acidity, respectively. In all cases, a humic substances concentration range 1.90 to 39.1 g L^−1^ was applied. The effect of time on humate retention was also investigated in a 2- to 180-minute range.

Desorption experiments were carried out in humic-loaded fly ash (0.5 g) by washing with distilled water (10 mL). The aqueous extract was centrifuged from fly ash and the concentration of the humic substances released in water was calculated spectrophotometrically.

For the determination of microorganisms the method of progressive dilution in liquid media by direct plating was used. In the case of bacteria specimens, 1g of the material tested was mixed with 10 mL Ringer solution (2.25 g L^−1^ NaCl, 0.10 g L^−1^ KCl, 0.12 g L^−1^ CaCl_2_·2H_2_O and 0.05 g L^−1^ NaHCO_3_). Nutrient agar was added as growing medium. The samples were incubated at 310 K for 48  hours prior to counting colonies. For the cultivation of fungi 1 g of the sample was mixed with 2 mL Ringer solution. Sabouraud was used as growing medium and X900 vials were also added to increase the selectivity. The samples were incubated at 303 K for 7 days prior to counting colonies and fungi identification. Two replicates per dilution were plated and counted. All materials and equipment used were sterile. Control cultures were also ran in the absence of the studied compounds. The microbial population was counted in cfu g^−1^ of the compound tested.

A Perkin-Elmer 883 IR spectrophotometer was employed to record infrared spectra; pellets of the specimens were prepared after mixing with dry KBr. The chemical analyses of fly ash samples were obtained from EDXRF measurements on a MDX1000 OXFORD spectrophotometer. Solids were separated from solutions with the use of an MLW T 54 centrifuge (15 minutes at 3600  minutes^ −1^). Acidity measurements were carried out by a SANXIN PHS-3D pH meter.

## 3. Results and Discussion

### 3.1. Parameters of Adsorption

**Figure 1:**
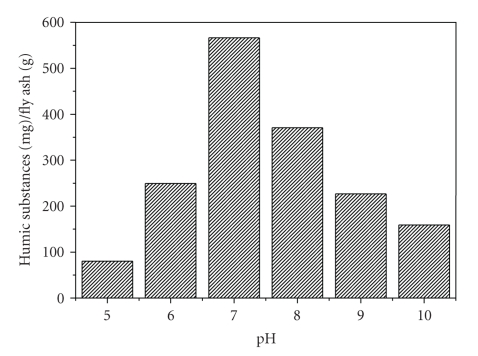
Retention of humic substances with pH.

The time of sorbent-adsorbate interaction and the acidity of the solution, both useful in setting up the adsorption process, are of great importance in adsorption phenomena. The retention of humic substances on fly ash increased only during the first 30  minutes and then remained practically constant; that is, equilibrium was attained. The solution acidity affects both the surface charge of the adsorbent and the protonation/deprotonation equilibria of the adsorbate. Figure 1 indicates that neutral humic solutions mostly favor the adsorption of humic substances onto fly ash. An increase in pH facilitates deprotonation of both fly ash and humic substances while at low pH values both sorbent and adsorbate become positively charged. Carboxylic groups, the principal source of charge development in natural organic matter, are ionized in pH = 4-5 [21]. Neutrality of humic substances solution seems to compensate the repulsive forces developed in homonymous charges in both host and guest materials, thus, favoring adsorption.

The effect of pH clearly shows that Coulombic interactions are involved in adsorption. However, the low cation exchange capacity of the Megalopolis fly ash (2.5 meq/100 g) cannot account for the whole amount of the adsorbate and, thus, insinuates additional dispersive host-guest interactions of hydrophobic nature (van der Waals, *π*-*π*, CH-*π*) and hydrogen bonds induced by the hydrophobic domains present in the humic superstructures [18].

### 3.2. Adsorbent Effect

Four different ashes (two fly ashes and two bottom ashes) from the Megalopolis and Ptolemais (Kardia) Basins were tested for the adsorption of humic substances. The chemical composition and properties of these ashes are presented in Table 1. Table 2 points out significant differences in adsorption of humic substances on these ashes. Specifically, both Megalopolis ashes retain more humic substances compared with the corresponding Kardia ones, a fact initially ascribed to the differences in the silicate content (Table 1).

**Figure 2:**
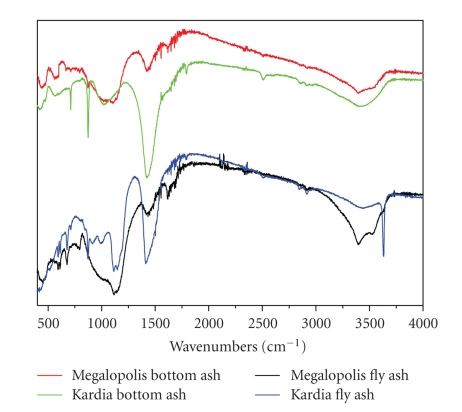
IR spectra of Megalopolis and Kardia ashes.

In order to explain this observation, IR spectra were received (Figure 2) to determine changes in structure additional to the changes in the chemical composition of the adsorbents. The main infrared bands are located at approximately 3450 cm^−1^ and 1640 cm^−1^ for O–H stretching and O–H bending, respectively [22]. The band at 1460 cm^−1^ represents the sodium carbonate resulting from carbonation [23]. 

**Figure 3:**
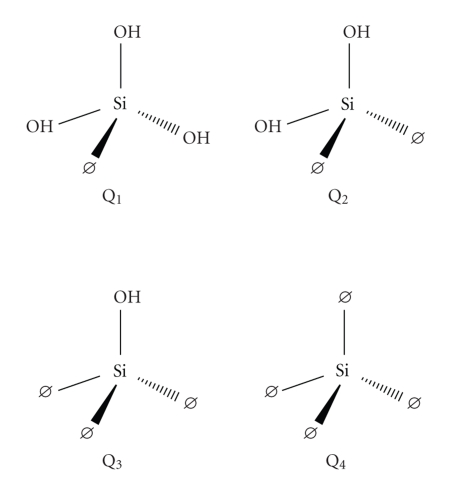
Schematic structures of silicate tetrahedral units, Q_*n*_, with *n* denoting the number of oxygen atoms bridging two silicon centers (*Ø* = bridging oxygen atom).

However, major differences are observed in the 850- to 1200- cm^−1^ envelope providing information on the silicate backbone. Particularly, the intense band at about 1090 cm^−1^ (accompanied by a shoulder at about 1175 cm^−1^) has been attributed to the asymmetric stretching vibrations of Si–O–Si bridges [22]. Such Si–O–Si bridges can be broken either by creation of nonbridging oxygen atoms in Si–O^−^ bonds (which are charge-compensated by metal ions) or by hydrolysis effects leading to the formation of Si–OH bonds in Q_*n*_ structures (Figure 3). The presence of nonbridging oxygen atoms leads to a weakening of the aluminosilicate network manifested as a pronounced shift towards lower frequencies in IR spectra. On this basis, the 850- to 1200-cm^−1^ envelope in both Megalopolis ashes is wider and more intense, particularly at lower wavenumbers, indicating a less “polymerized” silicate structure that readily adsorbs higher amounts of humic substances compared with the Kardia's corresponding ones, most probably via a cation exchange mechanism.

This explanation is fully compatible with the significant increase in adsorption of Kardia's bottom ash compared with the fly ash. The presence of intense absorption bands peaking at wavelengths lower than 1000 cm^−1^, in the case of the bottom ash, demonstrates changes in the matrix, that is, the existence of many nonbridging oxygen atoms responsible for humic adsorption.

### 3.3. Equilibrium Adsorption

**Figure 4:**
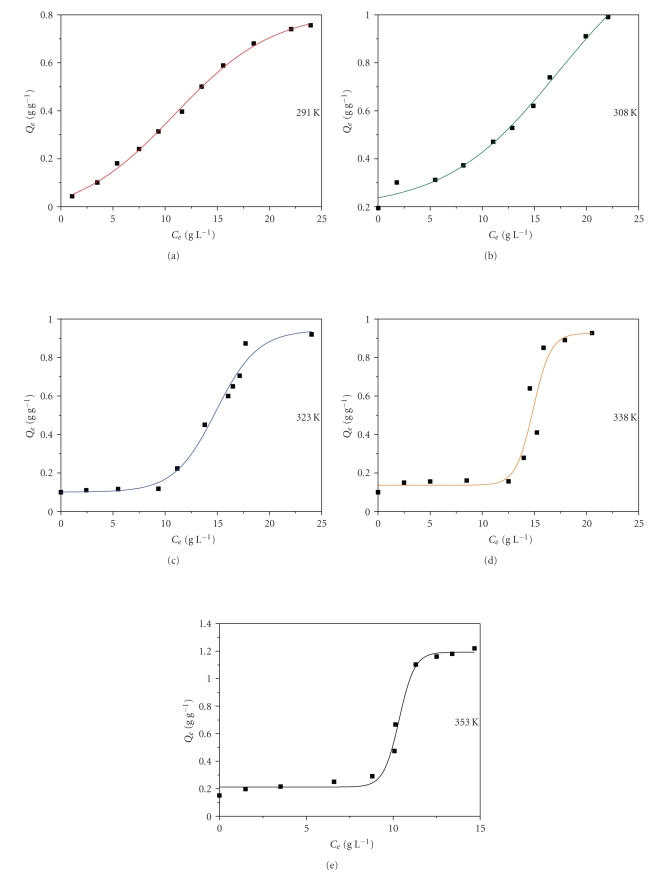
Adsorption of humic substances onto fly ash with temperature.

The values of equilibrium humic content, *Q*_*e*_, on fly ash as a function of the equilibrium humic concentration in solution, *C*_*e*_, at several temperatures (291, 308, 323, 338, and 353 K) are plotted in Figure 4. All isotherms appear sigmoidal, corresponding to a type II adsorption isotherm according to the Brunauer-Deming-Deming-Teller (BDDT) classification, this feature becoming more distinct with increasing temperature. Adsorption of humic substances onto fly ash ranges from 40 to 1300 mg humics/g fly ash at 291 and 353 K, respectively. Adsorption capacity increases with temperature from 760 to 1300 mg humics/g fly ash at 291 and 353 K, respectively (Figure 4). Endothermic adsorption processes usually indicate processes activated in a reaction-kinetic sense representing the combined effects of temperature on both the rate of approach to equilibrium and the position of the equilibrium itself. This encounters the surpassing of the energy barriers by the adsorbate to diffuse through narrow constrictions of the fly ash surface to cavities beyond. In addition, adsorption is accompanied by other subprocesses, for example, conformational changes, multiple equilibria, and retention mechanisms, which, when lumped together, contribute to the endothermic character of the adsorption. Thus, the “knee” in the adsorption isotherm represents an adsorption restart, possibly due to multilayer formation and/or the beginning of another adsorption subprocess.

### 3.4. Adsorption Isotherms

Five of the most valid adsorption models, that is, the Langmuir, Freundlich, BET, Harkins-Jura, and Dubinin-Radushkevich (DR) isotherm equations, were fit to the experimental data of humate adsorption onto fly ash particles. The aim was to reveal distinct physicochemical properties that characterize both the adsorbents and the sorption process. The Langmuir equation [24], although widely applied, cannot describe the adsorption adequately (*R* ^2^ < 0.67) in our case. This is not unexpected, since this theoretical model does not apply to Type II adsorption isotherms.

On the other hand, the Freundlich isotherm [25] provides a good mathematical model to describe the adsorption (Table 3) pinpointing heterogeneously distributed adsorption sites. Such an uneven arrangement of the heterogeneously adsorbed humates has been proved to promote the development of local multilayers and aggregates of the adsorbate [26].

The BET equation [27] describes adequately the adsorption behavior of humates onto fly ash particles, although this model tacitly assumes a homogeneous adsorption surface and gives a linear region only at the lower concentrations of adsorbate where a condensed film exists. On this basis, the applicability of the BET equation results from the existence of different homogeneous surface patches where adsorption occurs consecutively, so that the isotherm actually consists of an assortment of numerous steps each one corresponding to the adsorption occurring at these patches [26, 28].

The Harkins-Jura model [29] is mostly based on the assumption of a nonuniform distribution of the adsorption sites and is not applicable unless a condensed film is shaped. Compared with the BET theory the existence of a linear region is much more extensive involving higher adsorbate concentrations. However, the assembling of multilayers in the case of humate adsorption seems to meet better the BET theory requirements as the correlation coefficients of the Harkins-Jura equation summarized in Table 3 are lower.

The DR model [30] presupposes a Gaussian distribution of the adsorption space with respect to the adsorption potential. The adsorption is considered to be the result of the adsorbate molecule entering into a pore; the process is described according to the Polanyi comprehension of the energetics inside the empty space of solids and applies rather to structurally homogeneous systems. Although it is widely applied in Type I isotherms, this model fits quite satisfactorily the experimental data of humate adsorption onto fly ash (Table 3). This may be due to the facts that the DR equation does not reduce to Henry's law at low adsorbate concentrations, the linear region is also extensive, involving higher adsorbate concentrations, and that it applies at low adsorbent loadings. Van der Waals forces to account for host-guest interactions are also encountered.

From the DR plots, the mean energy of the adsorption, *E*, can be calculated using the following relationship:

(2) $$E=\frac{1}{\sqrt{2\beta }}.$$

The values of *E* do not exceed 0.2 kJ g^−1^ of adsorbate, accounting for strong forces of attraction, more intense than the Van der Waals forces.

### 3.5. Desorption Studies

**Figure 5:**
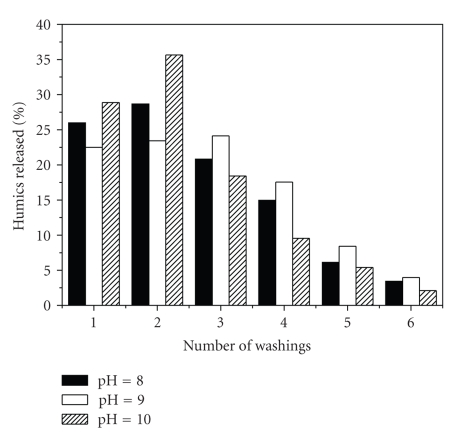
Desorption of humic substances by washing.

Desorption experiments were carried out to evaluate the ability of fly ash to render back the humic substances adsorbed, useful in potential applications of superficially modified fly ash as a slow-release fertilizer. Humic substances could not be released neither in acid nor in neutral environments; thus, experiments were processed in the alkaline area (pH = 8–10). Higher pH values were not tested because of their lack of importance for soil applications. The results presented in Figure 5 demonstrate that most of the humates (80%) were released during the first three washings; yet humics could be still identified after the sixth washing of fly ash. These observations clearly indicate that humic substances associate strongly with fly ash allowing for their slow release during repeated washing under alkaline conditions.

### 3.6. Microbial Populations

Microbial tests were carried out to determine the bacteria and fungi populations as the native microbial population in soil plays a vital role in the recycling of C, N, and P of the biosphere and, therefore, is critical for fertility and plant growth. Megalopolis fly ash proved sterile but humic-loaded samples produced 5 × 10^4^ bacteria. It is important to note that a fertile agricultural soil contains about 10^7^ and 5 × 10^5^ bacteria and fungi, respectively [31]. *Penicillium* spp, *Aureobasidium* spp, *Alternaria* spp, *Aspergilus* spp, *Mucor* spp, *Rhizopus* spp, *Rhizomucor* spp, and *Cladosporium* spp were identified, typical in soil and tissue degradation products. The population of fungi leveled the one found in a fertile agricultural soil. These observations annotate that fly ash partly favors the development of microbial life. This may be due to the fact that, as chemical analysis of trace elements shows, the Megalopolis fly ash (Table 4) contains metals that, in high concentrations, may be proved toxic to microbes restraining their growth. The presence of these trace elements causes no environmental risk as the increased pH of the Megalopolis fly ash (about 12) impairs the leaching of the metals [32]. However, it may set limitations regarding the mixing ratio of humic-loaded fly ash with soil in agricultural applications.

## 4. Conclusions

Humic-loaded fly ash was prepared at various humic to fly ash ratios. The Greek peaty lignite of the Megalopolis Basin was used as raw material for obtaining both fly ash and humates. Retention was studied at 291, 308, 323, 338, and 353 K and at several pH values. The adsorption capacity was found to increase at neutral pH and, also, with raise of temperature, that is, from 760 to 1300 mg humics/g fly ash at 291 K and 353 K, respectively. Fly ash demonstrated a high affinity towards humic substances and adsorption proceeded stepwise via strong Coulombic and hydrophophic forces of attraction between guest and host materials. Langmuir, Freundlich, BET, Harkins-Jura, and Dubinin-Radushkevich isotherm models were employed to evaluate the ongoing adsorption and shed light to the physicochemical properties of the sorbent-adsorbate system. Both the slow release of adsorbed humic substances during washing and the existence of microbial populations are considered advantageous for employing humic-loaded fly ash in biofunctional agricultural applications, that is, in biological cultivations substituting traditional soil-conditioning materials.

The importance of this study from an economic standpoint is emphasized by the advantageous preparation of humic-modified fly ash from power plant wastes. Its production could also prove profitable, thanks to the extended coal reserves worldwide, strongly supporting the viability of the research on this field.

## Figures and Tables

**Table 1:** Chemical analyses of fly ashes.

	Megalopolis		Kardia	
	Fly ash	Bottom Ash	Fly ash	Bottom Ash
SiO_2_^a^	39.6	41.7	23.0	17.8
Al_2_O_3_^a^	15.9	14.1	12.6	8.4
Fe_2_O_3_^a^	8.53	8.47	7.5	3.58
CaO^a^	18.3	24.6	44.7	29.9
MgO^a^	2.75	4.10	3.58	2.39
Na_2_O^a^	0.484	0.58	0.33	0.19
K_2_O^a^	1.72	1.79	2.20	0.40
SO_3_^a^	4.52	4.31	5.5	3.44
pH^b^	11.8	9.8	12.5	8.8

^a^% w/w,

^b^10% w/w in fly ash.

**Table 2:** Adsorption of humic substances on several bottom and fly ashes.

Adsorbent	Retention (g humic substances/g ash)
Fly ash	
Megalopolis	1.72
Kardia	0.62

Bottom ash	
Megalopolis	2.21
Kardia	1.31

**Table 3:** Correlation coefficients of the linear adsorption equations.

Isotherm equation	Temperature (K)	*R* ^2^
Langmuir *C*_*e*_/*Q*_*e*_ = 1/*K* *Q*_*m*_ + (1/*Q*_*m*_)*C*_*e*_	291	0.625
	308	0.608
	323	0.565
	338	0.445
	353	0.664

Freundlich log *Q*_*e*_ = log *K*_*F*_ + (1/*n*)log *C*_*e*_	291	0.989
	308	0.962
	323	0.871
	338	0.722
	353	0.759

BET *C*_*e*_/*Q*_*e*_(1 − *C*_*e*_) = 1/*X*_*m*_ *K*_*B*_ + ((*K*_*B*_ − 1)/*X*_*m*_ *K*_*B*_)*C*_*e*_	291	0.996
	308	0.929
	323	0.844
	338	0.880
	353	0.910

Harkins-Jura 1/*Q*_*e*_^2^ = *B*/*A* − (1/*A*)log *C*_*e*_	291	0.840
	308	0.969
	323	0.792
	338	0.520
	353	0.834

Dubinin-Radushkevich ln *Q*_*e*_ = ln *X*_*m*_ − *β*[*R* *T*ln (1+(1/*C*_*e*_))]^2^	291	0.957
	308	0.856
	323	0.985
	338	0.806
	353	0.893

**Table 4:** Trace elements concentrations in Megalopolis fly ash.

Substance	Concentration (ppm)
TiO_2_	0.823^a^
P_2_O_5_	0.233^a^
SrO	0.107^a^
BaO	680
MnO	620
Cr_2_O_3_	410
NiO	390
V_2_O_5_	340
ZnO	160
CuO	130
ZrO_2_	110
Rb_2_O	88
MoO_3_	73
PbO	58
Cl	43
Br	39
Y_2_O_3_	7

^a^% w/w.
